# Golgi Reassembly Stacking Protein 2 Modulates Myometrial Contractility during Labor by Affecting ATP Production

**DOI:** 10.3390/ijms241210116

**Published:** 2023-06-14

**Authors:** Fan Yang, Lina Chen, Bolun Wen, Xiaodi Wang, Lele Wang, Kaiyuan Ji, Huishu Liu

**Affiliations:** 1 School of Medicine, South China University of Technology, Guangzhou 510006, China; 2 Guangzhou Key Laboratory of Maternal-Fetal Medicine, Department of Obstetrics and Gynecology, Guangzhou Women and Children’s Medical Center, Guangzhou Medical University, Guangzhou 510623, China

**Keywords:** labor, myometrial contractility, *GORASP2*, ATP production, pregnancy

## Abstract

The mechanism of maintaining myometrial contractions during labor remains unclear. Autophagy has been reported to be activated in laboring myometrium, along with the high expression of Golgi reassembly stacking protein 2 (GORASP2), a protein capable of regulating autophagy activation. This study aimed to investigate the role and mechanism of GORASP2 in uterine contractions during labor. Western blot confirmed the increased expression of GORASP2 in laboring myometrium. Furthermore, the knockdown of *GORASP2* in primary human myometrial smooth muscle cells (hMSMCs) using siRNA resulted in reduced cell contractility. This phenomenon was independent of the contraction-associated protein and autophagy. Differential mRNAs were analyzed using RNA sequencing. Subsequently, KEGG pathway analysis identified that *GORASP2* knockdown suppressed several energy metabolism pathways. Furthermore, reduced ATP levels and aerobic respiration impairment were observed in measuring the oxygen consumption rate (OCR). These findings suggest that GORASP2 is up-regulated in the myometrium during labor and modulates myometrial contractility mainly by maintaining ATP production.

## 1. Introduction

In the myometrium, parturition manifests as forceful, synchronous, rhythmic contractions [1]. Regular uterine contractions have been known to contribute to safe delivery. However, the mechanism of myometrial contractions remains unclear. Previous studies on the regulation of uterine contraction focus on hormones, mechanical tension and hypoxia inflammation, etc. [2,3,4]. Our previous study reported that autophagy activation was observed in parturient myometrium, which could be associated with maintaining uterine contractions [5]. Autophagy also plays a role in maintaining pregnancy [6]. Studies reported that the deletion of *Becn1*, which was necessary for the fusion of autophagosomes and endosomes with lysosomes, in luteal cells inhibited autophagy and failed to maintain pregnancy in mice [7]. Moreover, during labor, the contraction of the myometrium was enhanced, and primary human myometrial smooth muscle cells (hMSMCs) were ischemic and hypoxic under this condition. Studies showed that hypoxia increased autophagy and enhanced contractions. Hypoxia induced autophagy and increased the expression of autophagy-related proteins ATG5, ATG7 and LC3b II in H9C2 cells [8,9]. Repetitive hypoxia treatment of pregnant rat myometrium and human myometrium increased the contraction of myometrium, indicating that hypoxia had a priming effect on myometrium [10].

The proteome of the laboring myometrium in humans revealed that Golgi Reassembly Stacking Protein 2 (GORASP2) was up-regulated in the laboring myometrium of humans compared to those not in labor [11]. *GORASP2* encodes GRASP55 and is mainly located in the Golgi apparatus [12]. When cells underwent glucose or amino acid deprivation, GORASP2 promoted autophagy by facilitating the fusion of LC3 of the autophagosome and LAMP2 of the lysosome [13,14,15]. When HeLa cells were treated with Earle’s Balanced Salt Solution (EBSS) to induce autophagy, knockdown of *GORASP2* significantly increased the levels of LC3b II and SQSTM1/p62, the accumulation of autophagosomes indicated that GORASP2 involved in autophagosome-lysosome fusion. In addition, when EBSS and BafA1 were used to treat bone marrow-derived macrophages simultaneously, *GORASP2* knockdown resulted in a reduction in the total number of autophagic puncta, suggesting that GORASP2 participated in autophagosome formation [16]. Therefore, we assumed that GORASP2 may play an important role in the regulation of myometrial contractions by mediating autophagy during labor.

Here, to explore the mechanisms of GORASP2 in myometrial contraction regulation, we aimed to knockdown *GORASP2* in human myometrial smooth muscle cells (hMSMCs) and detected cell contractility and autophagy under hypoxia. Additionally, we examined various biological processes of hMSMCs, such as ATP production, aerobic respiration, ROS production levels, apoptosis and cell cycle.

## 2. Results

### 2.1. Expression of GORASP2 Was Increased in the Myometrium during Labor

**Figure 1:**
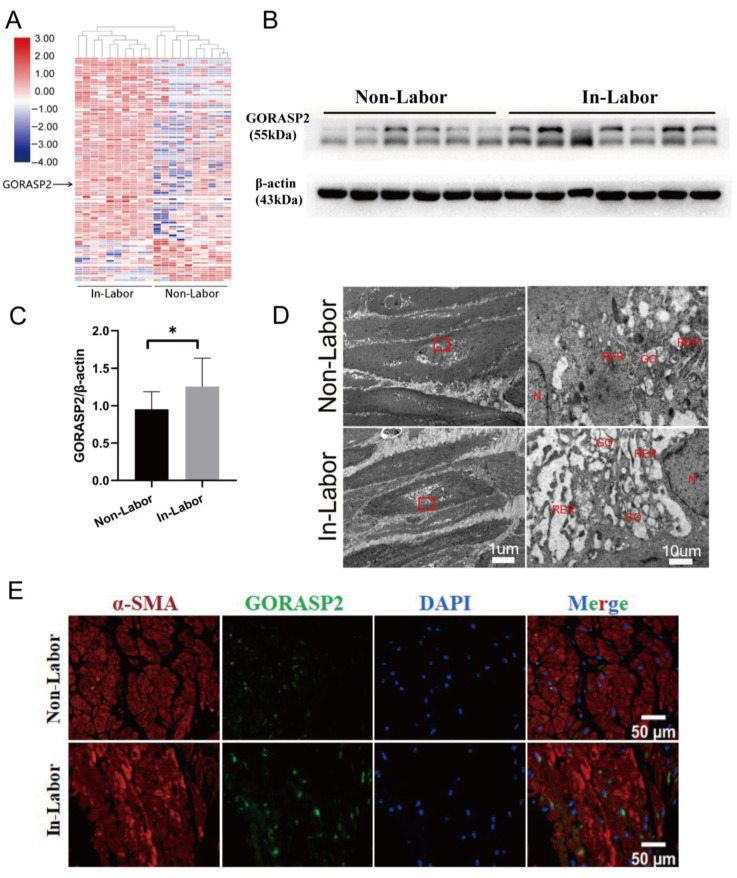
Golgi reassembly stacking protein 2 (GORASP2) is up-regulated in human in-labor myometrium. (**A**) Proteomics study comparing in-labor and non-labor myometrium. N = 10. (**B**,**C**) Expression of GORASP2 in in-labor and non-labor myometrium analyzed using Western blot. Non-Labor: N = 6, In-Labor: N = 7. (**D**) Electron microscopy images of human in-labor and non-labor myometrium. GO represents the Golgi apparatus; N indicates the nucleus; RER indicates the rough endoplasmic reticulum. (**E**) Visualization of GORASP2 in in-labor and non-labor myometrium via immunofluorescence, N = 3. * *p* < 0.05.

Based on the DIA proteomic data of the human myometrium in in-labor and non-labor [11], we found that the expression of GORASP2 was significantly higher in the myometrium during labor than in non-labor (Figure 1A). Western blot and immunofluorescence further confirmed that GORASP2 was highly expressed in in-labor myometrium and showed perinuclear distribution (Figure 1B,C,E). Furthermore, GORASP2 plays a critical role in maintaining the function and structure of the Golgi apparatus. To examine the morphological changes of the Golgi apparatus in the myometrium during labor, we performed transmission electron microscopy (TEM) detection, which revealed hypertrophy of the Golgi apparatus in the in-labor myometrium (Figure 1D), indicating active secretion or protein processing.

### 2.2. GORASP2 Was Involved in the Contractile Function of hMSMCs

**Figure 2:**
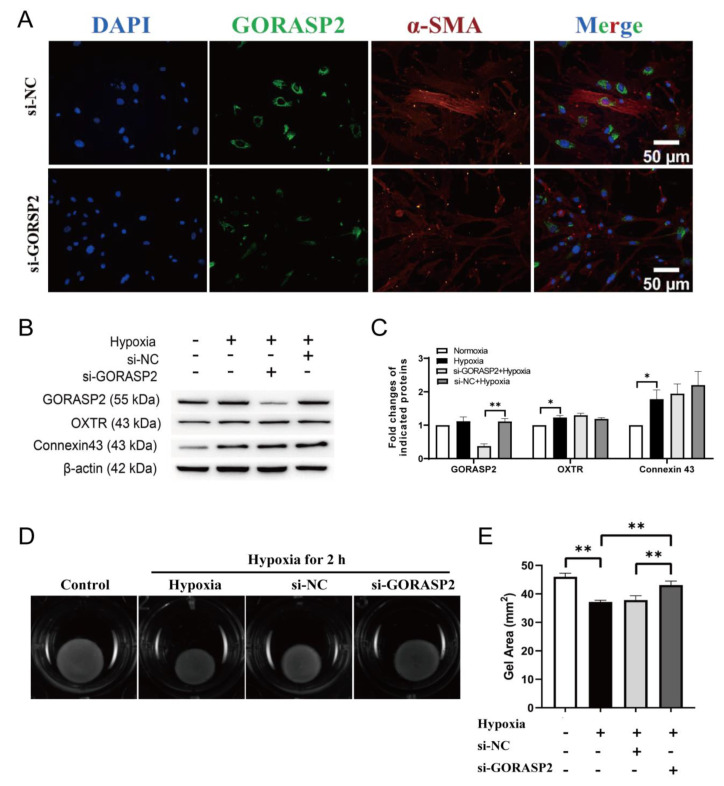
Knockdown of *GORASP2* decreased the contractility of primary human myometrial smooth muscle cells (hMSMCs). (**A**) hMSMCs were transfected with si-NC or si-*GORASP2* for 72 h, treated with 3% O_2_ for 2 h and stained for GORASP2, N = 3. (**B**,**C**) Expression of GORASP2, OXTR and Connexin 43 in hMSMCs treated with normoxia or 3% O_2_ for 2 h after transfection with si-NC or si-*GORASP2* for 72 h, N = 3. (**D**) Contractility assessment of hMSMCs transfected with si-NC or si-*GORASP2* for 48 h, treated with 10 nM oxytocin and 3% O_2_ for 2 h, N = 3; (**E**): Gel area statistics of hMSMCs contraction. * *p* < 0.05, ** *p* < 0.01.

Uterine smooth muscle cells are the main constituent of the myometrium, generating uterine contractions through a series of coupled physiological processes [17,18]. During labor, the hMSMCs experience hypoxic stress due to the transient ischemia of the myometrium caused by uterine contractions [19]. Therefore, in our subsequent cell experiments, we utilized hypoxic conditions to simulate this physiological scenario. The fluorescence signal of GORASP2 is primarily located in the perinuclear in hMSMCs and was reduced after *GORASP2* knockdown (Figure 2A). When hMSMCs were incubated under hypoxia, the well-known contraction-associated proteins, OXTR and Connexin 43, were up-regulated along with an increase in cell contractility (Figure 2B–D). Although the expression of GORASP2 showed no significant change under hypoxia and no influence on the expression of OXTR and Connexin 43, depletion of GORASP2 still partially reversed the cell contractility enhanced by hypoxia (Figure 2B–D). Moreover, a decrease in cell contractility due to *GORASP2* knockdown was also observed under normoxia conditions (Figure S1A).

### 2.3. GORASP2 on the Biological Functions of hMSMCs

**Figure 3:**
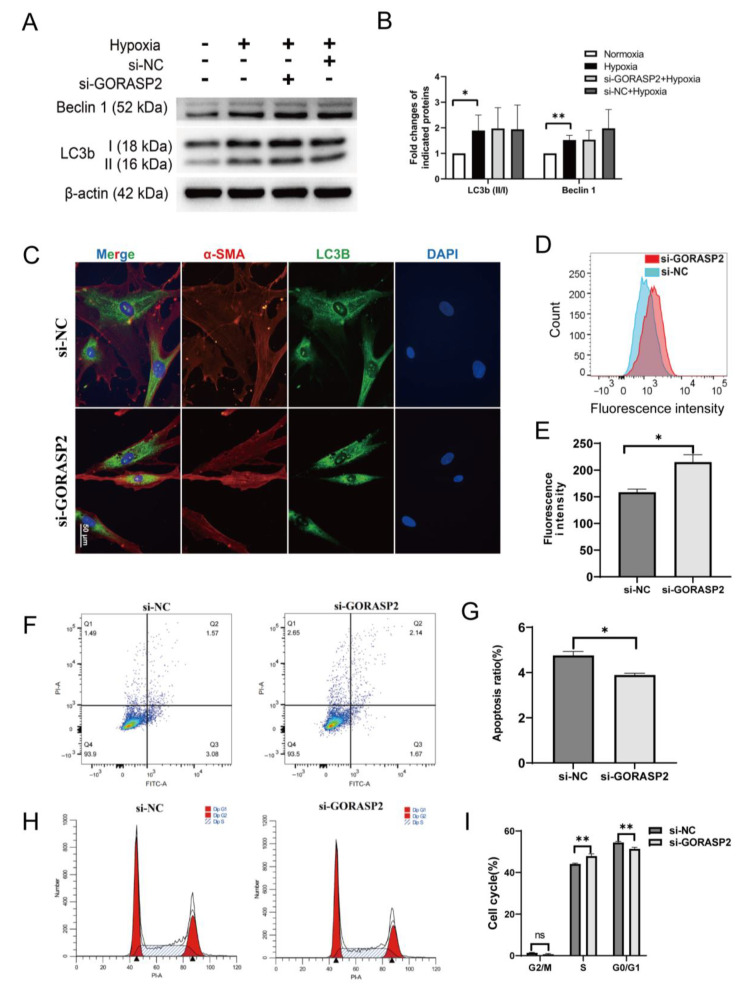
The effects of GORASP2 on the biological functions of hMSMCs. (**A**,**B**) Assessment of autophagy via the expression levels of LC3b II/I and Beclin 1 in hMSMCs treated with normoxia or 3% O_2_ for 2 h after transfected with si-NC or si-*GORASP2* for 72 h, N = 3. (**C**) Immunofluorescence of LC3b in hMSMCs treated with 3% O_2_ for 2 h after transfected with si-NC or si-*GORASP2* for 48 h, N = 3. (**D**,**E**) Intracellular reactive oxygen species (ROS) detected in hMSMCs treated with 3% O_2_ for 2 h after transfected with si-NC or si-*GORASP2* for 72 h, N = 3. (**F**,**G**) Apoptosis ratios in hMSMCs treated with 3% O_2_ for 2 h after transfected with si-NC or si-*GORASP2* for 72 h. The lower right quadrant (Q3, Annexin V+/PI-) represents early apoptotic cells, the upper right quadrant (Q2, Annexin V+/PI+) represents late apoptotic cells and necrotic cells, and the upper left quadrant (Q1, Annexin V-/PI+) is considered to be within the scope of the assay error. The total amount of Q2 and Q3 were analyzed between the groups. N = 3. (**H**,**I**) The cell cycle of hMSMCs treated with 3% O_2_ for 2 h after transfected with si-NC or si-*GORASP2* for 72 h. The levels of G1, S and G2 phases were analyzed between the groups; N = 3. ns, no statistical difference, * *p* < 0.05, ** *p* < 0.01.

The expression levels of autophagy-related proteins, such as light chain 3 beta (LC3b) and Beclin 1, were detected using Western blot. The results showed that hypoxia induced autophagy in hMSMCs. However, *GORASP2* knockdown failed to inhibit this phenomenon (Figure 3A,B). Additionally, there was no significant difference in the LC3b fluorescence signal observed in the cytoplasm of hMSMCs after *GORASP2* knockdown compared to before knockdown (Figure 3C). Rapamycin effectively induced autophagy in hMSMCs (Figure S1B), but *GORASP2* knockdown did not change autophagy levels under rapamycin treatment (Figure S1C,D). We further explored the viability of hMSMCs after *GORASP2* knockdown using flow cytometry. The knockdown of *GORASP2* in hMSMCs resulted in an increase in intracellular ROS (Figure 3D,E) and apoptosis ratio (Figure 3F,G), and the cell cycle was arrested in the S phase (Figure 3H,I).

### 2.4. GORASP2 Maintained the Energy Metabolism Levels of hMSMCs

**Figure 4:**
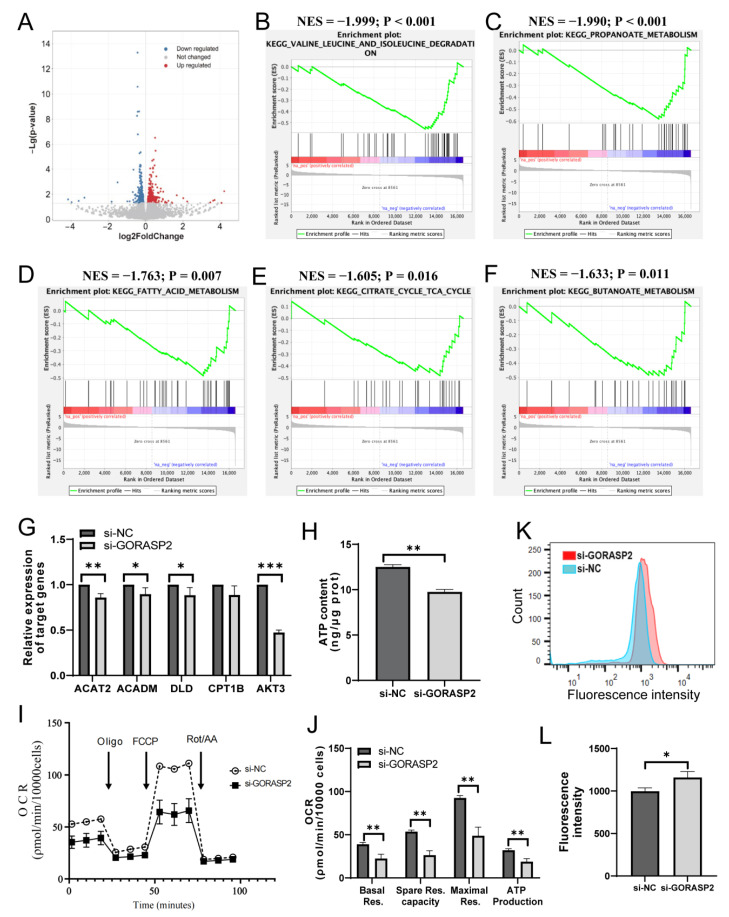
The effects of GORASP2 on energy production in hMSMCs. (**A**) Volcanic plots of RNA expression treated with 3% O_2_ for 2 h after transfected with si-NC or si-*GORASP2* for 48 h, N = 3. (**B**–**F**) KEGG-enriched pathways via Gene Set Enrichment Analysis reveal five significantly downregulated pathways related to metabolism. (**G**) Validation of genes related to the energy metabolism pathways using RT-qPCR, N = 3. (**H**) ATP levels in hMSMCs treated with 3% O_2_ for 2 h after transfected with si-NC or si-*GORASP2* for 72 h. The ATP level (ng) was normalized with the protein amount (μg Prot) obtained from the groups, N = 3. (**I**,**J**) Oxygen consumption rate (OCR) of hMSMCs treated with 300 μM CoCl_2_ for 2 h after transfected with si-NC or si-*GORASP2* for 72 h. Subsequent additions of ATP synthase inhibitor oligomycin, uncoupler FCCP, ETC complex I inhibitor rotenone and respiratory complex III inhibitor antimycin A were performed. Basal respiration, ATP-production coupled respiration, maximal respiration and spare respiratory capacity were calculated from the OCR trace, N = 3. (**K**,**L**) Mitochondrial ROS detected in hMSMCs treated with 3% O_2_ for 2 h after transfected with si-NC or si-*GORASP2* for 72 h, N = 3. * *p* < 0.05, ** *p* < 0.01, *** *p* < 0.001.

To further determine the role of GORASP2 in the contractile function of hMSMCs under hypoxia, RNA-sequencing was employed on hMSMCs with *GORASP2* knockdown (Figure 4A). The KEGG pathways database was used for enrichment analysis. The results showed that among the top 20 downregulated pathways, five were associated with metabolism, namely valine, leucine and isoleucine degradation, propanoate metabolism, fatty acid metabolism, citrate cycle TCA cycle and butanoate metabolism (Figure 4B–F). To further verify that *GORASP2* knockdown suppressed the expression of genes related to metabolism and mitochondria, the abundances of indicated genes, such as dihydrolipoamide dehydrogenase (*DLD*), carnitine palmitoyltransferase 1B (*CPT1B*), AKT Serine/Threonine Kinase 3 (*AKT3*), acetyl-CoA Acetyltransferase 2 (*ACAT2*) and acyl-CoA dehydrogenase medium chain (*ACADM*) were evaluated in hMSMCs after *GORASP2* knockdown. The results revealed that the mRNA levels of *ACAT2*, *AKT3*, *ACADM* and *DLD* were downregulated in hMSMCs after *GORASP2* knockdown (Figure 4G).

The intense contraction caused by myometrium during labor is a highly energy-consuming process [20], and energy supply is essential for hMSMCs contraction [21]. To demonstrate the impact of GORASP2 on metabolism under hypoxic stress, the intracellular ATP of hMSMCs was evaluated. The results revealed that ATP levels significantly decreased in hMSMCs after the knockdown of *GORASP2*, with a decline of 21.9% (Figure 4H). Subsequently, the OCR of hMSMCs was determined. Compared to the si-NC group, basal respiration was decreased in the si-*GORASP2* group. After the inhibition of the oxidative respiratory chain using oligomycin, the OCR of the two groups decreased significantly, which was regarded as ATP-production coupled respiration. Furthermore, the maximal respiration elicited by carbonylcyanide-4-(trifluoromethoxy) phenylhydrazone (FCCP) was significantly decreased on *GORASP2* knockdown, resulting in reduced spare respiratory capacity in hMSMCs (Figure 4I,J). To further clarify the origin of the increased intracellular ROS (Figure 3D,E), an additional assessment of mitochondrial ROS by using MitSOX dyes was performed and showed an increase in mitochondrial ROS resulting from the knockdown of *GORASP2* (Figure 4K,L).

## 3. Discussion

This study revealed that GORASP2 was up-regulated in the laboring myometrium. The knockdown of *GORASP2* under hypoxia affected the biological functions of hMSMCs, such as the response for ROS, cell cycle, apoptosis and cell contractility; however, autophagy activation was not affected. The transcriptomes altered by the knockdown of *GORASP2* in hMSMCs were mainly enriched in metabolism-related pathways, which were validated via OCR detection.

The mechanism of GORASP2 facilitating autophagosome-lysosome fusion has been well-reported in HeLa and BMM cells [14,16]. However, the knockdown of *GORASP2* in hMSMCs under hypoxia neither influenced autophagosome morphology nor reduced LC3b II/I ratio and Beclin1 expression, indicating that GORASP2 was not involved in hypoxia-induced autophagy in hMSMCs. This discrepancy could be attributed to differences in the origin of the experimental cells and experimental conditions. Moreover, studies have shown that GORASP2 de-O-GlcNAcylation under glucose starvation is required for GORASP2 targeting autophagosomes [13]; hypoxia may not trigger changes in GORASP2 protein modification. Although our previous study revealed the activation of autophagy in the laboring myometrium [5], it is not significantly regulated by GORASP2, at least not under hypoxic conditions.

After *GORASP2* was knocked down in hMSMCs, cell contractility decreased. As GORASP2 was not observed to be involved in the autophagy process in hMSMCs, we investigated other possible mechanisms regulating cell contractility. GORASP2 is directly involved in maintaining the structure and function of the Golgi apparatus, playing a role in cisternae stacking, mitosis, apoptosis, cell migration and cargo secretion [22,23,24,25]. Accordingly, *GORASP2* knockdown has been reported to fragment the structure of the Golgi apparatus [26]. Additionally, GORASP2 plays an important role in cell cycle regulation via the MEK1/ERK pathway [27]. Our results showed that *GORASP2* knockdown increased the level of ROS and apoptosis, and arrested the cell cycle in the S phase, indicating GORASP2 involvement in hMSMCs’ biological processes, despite the changes minor.

Notably, RNA-sequencing and KEGG pathways analysis revealed that *GORASP2* knockdown suppressed energy metabolism pathways. *GORASP2* knockdown also reduced ATP production levels of hMSMCs and impaired aerobic respiration. During labor, the energy metabolism of the myometrium is up-regulated to support sufficient energy for intense contraction [21,28,29]. Insufficient ATP supply during labor could decrease uterine contractility, causing uterine atony, prolonged labor, failure to progress or postpartum hemorrhage, etc. [30,31,32,33]. Studies have shown that application of extracellular ATP significantly increased the force of spontaneous contraction in uterine strips of term-pregnant rats and humans [34,35,36].

As GORASP2 is not a protein directly involved in the energy metabolism pathway, it is inferred that it may regulate the expression of metabolism-related genes. RT-qPCR confirmed that several metabolism-related genes (*ACAT2*, *ACADM*, *DLD* and *AKT3*) were down-regulated after *GORASP2* knockdown. *ACADM* encodes medium-chain acyl-CoA dehydrogenase, which catalyzes the first step of β-oxidation and handles the breaking down of medium-chain fatty acids. Moreover, *ACADM* mutation can induce metabolic diseases [37]. *DLD* encodes dihydrolipoamide dehydrogenase and is an essential mitochondrial enzyme for energy metabolism and redox balance across eukaryotes [38]. AKT3 is a member of the AKT, serine/threonine protein kinase family; knockdown of *AKT3* induces mitochondrial dysfunction and decreases levels of mitochondrial biogenesis [39,40]. Mitochondria are organelles that produce respiratory ATP in eukaryotes [41]. Mitochondrial damage can lead to an energy metabolism disorder [42]. Our results showed that *GORASP2* knockdown significantly decreased *AKT3* expression and increased mitochondrial ROS, indicating *AKT3* is probably a key downstream target of *GORASP2*, which is important for maintaining mitochondrial function and metabolism pathways in hMSMCs. Further research on the potential roles of the *GORASP2-AKT3* axis in oxidative phosphorylation, mitochondrial function, and cell contractility would help our understanding of the underlying mechanisms. 

In addition, this study provided evidence that GORASP2 affects energy generation in hMSMCs; we also found several metabolic pathways related to GORASP2, such as amino acid metabolism and fatty acid metabolism. Our previous untargeted metabolomic analysis found that lipolysis and fatty acid oxidation were most significantly increased in in-labor myometrium, compared with non-labor myometrium [21], indicating metabolic changes during labor. The specific metabolic pathway that is actually relevant to GORASP2 needs further experiments.

In summary, this study identified that GORASP2 modulates myometrial contractility mainly by participating in the regulation of energy metabolism pathways. This finding is helpful in promoting the development of new strategies for monitoring uterine contraction and guiding energy supplementation during labor. Further research is required to explore the mechanism of ATP regulation in myometrial contractility.

## 4. Materials and Methods

### 4.1. Antibodies

Anti-α-SMA (ab7817, Abcam, Cambridge, UK), anti-GORASP2 (ab204335, Abcam, Cambridge, UK), anti-LC3b (L7543, Sigma-Aldrich, St. Louis, MO, USA), Alexa 594 goat anti-mouse secondary antibody (ab150116, Abcam, Cambridge, UK) or Alexa goat anti-rabbit 488-IgG (ab150077, Abcam, Cambridge, UK), anti-oxytocin receptor (ab181077, Abcam, Cambridge, UK), anti-Connexin 43 (#3512, Cell Signaling Technology, Beverly, MA, USA), anti-Beclin 1 (ab62557, Abcam, Cambridge, UK) and anti-β-actin (AF7018, Affinity, San Francisco, CO, USA).

### 4.2. Subjects and Tissue Collection

Myometrial biopsies were obtained from women during cesarean sections at Guangzhou Women and Children’s Medical Center. The inclusion criteria were as follows: term, singleton and primiparas, without any medical complications (such as hypertension, eclampsia, cholestasis or gestational diabetes) or abnormal labor (such as uterine atony or prolonged labor). Labor was defined as regular palpable contractions and cervical dilation. The tissues were cut from the upper edge of the lower uterine incision as a sample, washed with cold phosphate-buffered saline (PBS) at 4 °C immediately to remove blood, and the attached decidua, after the tissue samples were quickly frozen in liquid nitrogen and then stored at −80 °C. This study was approved by the Ethics Committee of the Guangzhou Women and Children Medical Center (No. 2023040A01).

### 4.3. Western Blot

Protein was extracted from myometrium and hMSMCs using RIPA lysis buffer containing protease inhibitor cocktail (P0044-1ML, Sigma-Aldrich, St. Louis, MI, USA). Protein concentration was measured using a BCA assay kit (#23227, Thermo Scientific, Watham, MA, USA). Western blot protein samples were loaded in SDS-PAGE gel, separated by electrophoresis, and transferred onto PVDF (polyvinylidene fluoride) membranes (IPVH00010, Millipore, Darmstadt, Germany). Protein levels were quantified by a ChemiDoc XRS+, and the gray value of the target protein was calculated by Image J (V1.8.0.112, NIH, Bethesda, MD, USA). 

### 4.4. Transmission Electron Photography

Fresh myometrial tissues were cut into 1 mm^3^ sections, or cells (1 × 10^7^) were collected with trypsin and centrifuged. Then the samples were fixed with an electron microscope fixing solution (0.1 M sodium cacodylate, PH 7.4) at 4 °C. The samples were washed thrice with 0.1 M phosphate buffer (PH 7.4) for 45 min. Afterward, the samples were dehydrated using graded ethanol (30–100%) and 100% acetone at room temperature. The samples were embedded in EMBed 812 (90529-77-4, SPI, Guangzhou, China) at 37 °C overnight. Then, the embedding plates were heated at 60 °C for 48 h to complete polymerization. Following this, the resin blocks were cut into slices at 60 to 80 nm with an ultrathin slicer (LEICA EM UC7, Wetzlar, Germany). A total of 2% uranium acetate saturated alcohol solution was used to avoid lightly staining for 8 min. After this, the samples were rinsed thrice in 70% ethanol and then in ultra-pure water. The samples were stained by lead citrate (2.6%) for 8 min in a CO_2_-free environment and then rinsed thrice with ultra-pure water. After the cuprum grids were dried overnight, the images were visualized under a transmission electron microscope (HT7800/HT7700, HITACHI, Tokyo, Japan).

### 4.5. Primary Culture and hMSMCs Identification

Myometrial tissues were cut into small pieces and evenly spread into culture dishes with Dulbecco’s Modified Eagle Medium (DMEM) containing 10% fetal bovine serum (FBS, #10099-141, Gibco, Grand Island, NY, USA) and 1% penicillin-streptomycin (10,000 U/mL, #15140122, Gibco, Grand Island, NY, USA), cultured at 37 °C, 5% CO_2_. When the cells crawled out of the tissue, the positive marker α-SMA was used to identify uterine smooth muscle cells by immunofluorescence.

Hypoxic treatment for hMSMCs was described in our previous study [43], under the condition of 3% O_2_, 5% CO_2_ and 92% N_2_ for 2 h in a hypoxia incubator (Baker Ruskinn Technology, Ltd., South Wales, UK).

### 4.6. Immunofluorescence

The paraffin-embedded sections were dewaxed with graded alcohol and then subjected to antigen retrieval with sodium citrate buffer. For cells, they were fixed in methanol for 20 min and washed thrice with PBST (0.3% Triton X-100 in PBS). The sections or cells were blocked using 10% goat serum at 37 °C for 1 h and then incubated successively with target antibodies at 4 °C overnight and secondary antibodies for 1 h at 37 °C in darkness. The images were visualized under Leica DMi8 fluorescence microscopy (DMi8, Leica, Wetzlar, Germany).

### 4.7. Cell Transfection

GORASP2 was silenced using siRNA (si-*GORASP2*, F: 5′-GGCUGGUACACCUAUUACA-3′; R: 5′- UGUAAUAGGUGUACCAGCC-3′). The cells were transfected with si-*GORASP2* or the control siRNA (si-NC, F: 5′-UUCUCCGAACGUGUCACGU-3′; R: 5′-ACGUGACACGUUCGGAGAA-3′) with a final concentration of 10 nM using the transfection reagent (INTERFERin^®^, 409-10, Polyplus, Strasbourg, France) according to the manufacturer’s protocol. Cells were harvested for subsequent experiments after 48–72 h transfection.

Autophagy flux was detected using LC3b overexpression adenovirus with green and red fluorescence protein tags (RFP-GFP-LC3b) (TSB208221, TranSheep Biotechnology, Shanghai, China). hMSMCs were incubated with RFP-GFP-LC3b adenovirus for 2 h. Then the cells were cultured for an additional 48 h and treated with 20 μM rapamycin (AY22989, Sellseck, Houston, TX, USA) for 4 h before being photographed using a DMi8 fluorescence microscope (DMi8, Leica, Wetzlar, Germany).

### 4.8. Cell Contraction Assay

The contractility of hMSMCs was evaluated using a cell contraction kit (CBA-201, Cell Biolabs Inc. San Diego, CA, USA) according to the manufacturer’s instructions. Collagen solution was prepared and mixed with cell suspension, then added to a 24-well plate and cultured for 1 h to form a solid gel. Then, 1 mL of medium was added, and the cells were cultured for an additional 48 h. The hMSMCs were placed in either a hypoxic or normoxic environment and stimulated with oxytocin (10 nM at final concentration) to induce contractions. After 2 h of treatment, the gel was released by gently scraping along the well walls with a tip of a sterile pipette. The area of the gel was measured at 1–4 h using a ChemiDoc XRS+ (Bio-Rad, Hercules, CA, USA), and the value of gel area was calculated by Image Lab.

### 4.9. Reactive Oxygen Species and Apoptosis Assay

The hMSMCs were stained with H2DCFDA (D399, Thermo Scientific, MA, USA) or mtSOX Deep Red (MT14, Dojindo, Shanghai, China) to measure the intracellular ROS or mitochondrial ROS, respectively. The dyes’ final concentrations were 5 µM for H2DCFDA and 10 µM for mtSOX. After staining for 30 min, cells were rinsed thrice with PBS, flow cytometry (BD Accuri C6, Franklin Lakes, NJ, USA) and FlowJo 10.8.1 software (FlowJo, Ashland, OR, USA) was used for fluorescence analysis. For cell apoptosis detection, cells were collected and stained with Annexing V–FITC and propidium iodide solution (MultiSciences Biotech Co., Ltd., Hangzhou, China) for 15 min and analyzed using flow cytometry (BD Accuri C6, Franklin Lakes, NJ, USA) and FlowJo 10.8.1 software (FlowJo, Ashland, OR, USA).

### 4.10. Cell Cycle Detection

The cells were collected and rinsed thrice with PBS. After the cells were stained with a mixture of DNA staining solution and permeabilization solution for 30 min at room temperature in the dark; flow cytometry was used for detection.

### 4.11. RNA Sequencing (RNA-Seq) and Data Analysis

The RNA-seq library was generated using the VAHTS Universal V6 RNA-seq Library Prep Kit for Illumina^®^ (NR604-01/02). Briefly, mRNA was purified from total RNA using poly-T oligo-attached magnetic beads and then fragmented using a fragmentation buffer. Random hexamer primer and RNase H were used to synthesize the first strand of cDNA. Following this, buffer, dNTPs, DNA polymerase I and RNase H were used to synthesize the second strand of cDNA. The double-stranded cDNA was repaired at the end, underwent an addition of a tail and was connected to the sequencing connector. The final cDNA library was obtained using polymerase chain reaction (PCR). The RNA concentration of the library was measured using a Qubit^®^ RNA Assay Kit in Qubit^®^ 3.0 to preliminary quantify it and then dilute it to 1 ng/μL. Additionally, the insert size was assessed using the Agilent Bioanalyzer 2100 system (Agilent Technologies, Santa Clara, CA, USA). Quantitative PCR was used to accurately quantify the effective library concentration (>10 nm). The RNA-seq was carried out at Annoroad Gene Technology Company. The raw data is available in the Genome Sequence Archive repository (accession number HRA003447).

The RNA-seq reads were mapped to the human genome (H. sapiens, GRCh38) and transcriptome (Ensembl, release 84) using hisat2 with the default parameters. The read counts were calculated using feature counts, and Ensemble gene annotations (H. sapiens, release 84) were assigned to the reads. The mRNAs that were differentially expressed between the groups were considered significant at a *p*-value < 0.05, which was identified using the DEseq2 package of R. The Kyoto Encyclopedia of Genes and Genomes (KEGG) pathways were analyzed using Gene Set Enrichment Analysis (GSEA) in GSEA Java implementation (v4.1.0). The enrichment magnitude and statistical significance were quantified by normalized enrichment score (NES) and *p*-value [44]. The *p*-value < 0.05 was considered significant.

### 4.12. Reverse Transcription Quantitative Polymerase Chain Reaction (RT-qPCR)

The total RNA of hMSMCs was extracted and purified using a RNeasy Plus Mini Kit (#74136, QIAGEN, Shanghai, China). The RNA concentration was measured using Multiskan GO, and cDNA was synthesized using a reverse transcription kit (#RR047A, Takara, Otsu, Shiga, Japan). Real-time PCR was performed using TB Green Premix Ex Taq II (Tli RNaseH Plus, Takara, Otsu, Shiga, Japan) by QuantStudio™ Real-Time PCR System (Applied Biosystems, Foster City, CA, USA), with the temperature of 95 °C for 30 s, followed by 40 cycles of 95 °C for 15 s, then 60 °C for 30 s. The primer sequences were as follows: *DLD* (F: 5′-ATTGGCCGACGACCCTTTA-3′; R: 5′GCCTTCATCCTCTGCTTTGTG-3′), *ACADM* (F: 5′-ATGTGGATAACCAACGGAGGAAA-3′; R: 5′-CTAGTATCTGAACATCGCTGGCC-3′), *CPT1B* (F: 5′-AATCCTACTCCTATGACCCCGAA-3′; R: 5′-CCATTCTTGAAGGAAATGAGAGTG-3′), *ACAT2* (F: 5′-CTGTGGGCAGAATCCTGTTAGA-3′; R: 5′-GTGGCATCTCACCTATCTTTACTCC-3′), *AKT3* (F: 5′-CGGAAAGATTGTGTACCGTGATC-3′; R: 5′ CTTCATGGTGGCTGCATCTGTG-3′). The relative mRNA expression was calculated based on the 2^−ΔΔCT^ method, and tubulin beta 3 class III (*TUBB3*) was used as an endogenous control.

### 4.13. Mitochondrial Stress Assay

Cellular mitochondrial stress was monitored using the Seahorse Bioscience Extracellular Flux Analyzer (XF24, Seahorse Bioscience Inc., North Billerica, MA, USA) by measuring OCR, which is indicative of respiration in real-time. Briefly, 16,000–20,000 cells were seeded in 24-well plates designed for XF24 in 250 μL of appropriate growth media and incubated overnight. Prior to measurements, cells were treated with 300 μM CoCl_2_ for 2 h and then washed with unbuffered media once and immersed in 500 μL unbuffered media. Cells were sequentially treated as indicated with oligomycin (Oligo, 1.5 μM), carbonyl cyanide-4-(trifluoromethoxy) phenylhydrazone (FCCP, 2.0 μM) and rotenone (Rot/AA, 0.5 μM). The OCR was then measured in a typical eight-minute cycle of mix (two to four min), dwell (two min) and measure (two to four min) following the manufacturer’s instructions. Basal levels of OCR were recorded first, followed by the OCR levels after the injection of compounds that inhibit the respiratory mitochondrial electron transport chain and ATP synthesis. After performing the assay, each well was gently washed thrice with PBS and fixed with methanol. Data were normalized by cell counting using crystal violet staining.

### 4.14. ATP Detection Assay

ATP concentration in hMSMCs was determined using an ATP Colorimetric Assay Kit (#MAK190, Sigma-Aldrich, St. Louis, MI, USA). Briefly, hMSMCs (1 × 10^6^ cells per group) were lysed in 100 μL of ATP assay buffer. The lysates were deproteinized using a 10 kDA MWCO spin filter (14,000 rpm, 40 min, 4 °C), and the filtrates were collected for ATP assay, while concentrates were harvested for protein amount determination. The ATP standards and colorimetric reaction mix were prepared according to the manufacturer’s instructions. A total of 50 μL of samples and standards were added into wells of a 96-well plate, and 50 μL of reaction mix was applied to each well. The plate was incubated at room temperature for 30 min in a dark environment. Then, the absorbance was measured at 570 nm. Additionally, 10 μL of concentrates from the groups were diluted with 90 μL of ATP assay buffer, and the protein content was detected using a BCA assay kit. The ATP content was normalized with the detected protein and presented as ng/μg Prot.

### 4.15. Statistical Analysis

Data for continuous variables are presented as mean ± the standard error of the mean. Statistical analysis was performed using SPSS 13.0 (SPSS, Chicago, IL, USA) and GraphPad Prism 8.4.0 software (San Diego, CA, USA). The Student’s *t*-test was used to compare two independent groups and one-way ANOVA was used to compare three independent groups. Statistical significance was set at *p* < 0.05.

## Data Availability

The data presented in this study are openly available in the GSA database (reference number: HRA003447; https://ngdc.cncb.ac.cn/gsa-human/browse/HRA003447 (accessed on 8 May 2023)). All the data generated or analyzed during the current study are available in the Supplementary Information Files or from the corresponding author on reasonable request.

## Supplementary Materials

The supporting information can be downloaded at: https://www.mdpi.com/article/10.3390/ijms241210116/s1.
