# Supplementary material for: Golgi Reassembly Stacking Protein 2 Modulates Myometrial Contractility during Labor by Affecting ATP Production
Source: Int J Mol Sci. 2023 Jun 14;24(12):10116. doi: 10.3390/ijms241210116 (PMC10298969; doi:10.3390/ijms241210116)
Supplement: Supplementary file 1 [file ijms-24-10116-s001.zip › ijms-2415234-supplementary.pdf]

Figure S1

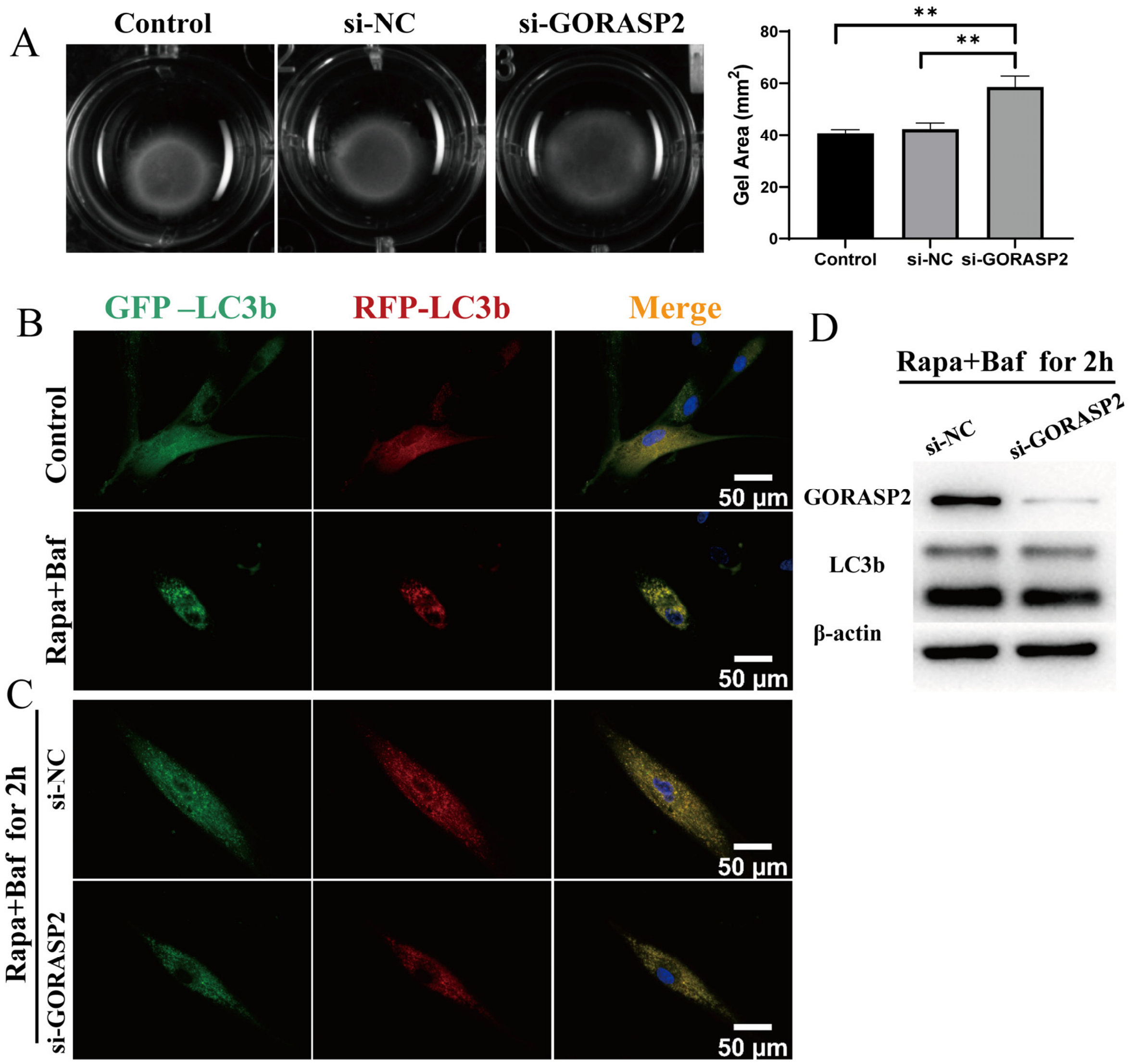

Figure S1. GORASP2 was involved in the contractile function of hMSMCs, but GORASP2 deficiency didn't affect the autophagy initiation of hMSMCs.

(**A**) Contractility assessment of hMSMCs transfected with si-NC or si-*GORASP2* for 48 h, treated with 10 nM oxytocin under normoxia for 2 h, N = 3. (**B**) hMSMCs were transfected with RFP–GFP–LC3 adenovirus for 2 h, treated with growth medium (Control) or Rapamycin+Bafa1 for 2 h, and detected the autophagy flux, N = 3. (**C**) hMSMCs were transfected with si-NC or si-*GORASP2* for 48 h, following transfected with RFP–GFP–LC3 adenovirus for 2 h, then treated with Rapamycin+Bafa1 for 2 h, and detected the autophagy flux, N = 3. (**D**) Western blot results for GORASP2 and LC3b in hMSMCs treated with Rapamycin+Bafa1 for 2 h after transfected with si-NC or si-*GORASP2* for 72 h, N = 3. **P < 0.01.
